# Strategic use of resources to enhance colorectal cancer screening for patients with diabetes (SURE: CRC4D) in federally qualified health centers: a protocol for hybrid type ii effectiveness-implementation trial

**DOI:** 10.1186/s12875-024-02496-0

**Published:** 2024-07-05

**Authors:** Denalee M. O’Malley, Benjamin F. Crabtree, Srivarsha Kaloth, Pamela Ohman-Strickland, Jeanne Ferrante, Shawna V. Hudson, Anita Y. Kinney

**Affiliations:** 1 Department of Family Medicine and Community Health, Research Division, Rutgers Robert Wood Johnson Medical School, 303 George Street, Rm 309, New Brunswick, NJ USA; 2 Rutgers Cancer Institute of New Jersey, New Brunswick, NJ USA; 3 Department of Biostatistics and Epidemiology, School of Public Health, Rutgers University, New Brunswick, NJ USA

**Keywords:** Colorectal cancer, Federally qualified health center, Diabetes mellitus, Hybrid 2 effectiveness-implementation, Implementation planning, EPIS, Primary care safety-net

## Abstract

**Background:**

Persons with diabetes have 27% elevated risk of developing colorectal cancer (CRC) and are disproportionately from priority health disparities populations. Federally qualified health centers (FQHCs) struggle to implement CRC screening programs for average risk patients. Strategies to effectively prioritize and optimize CRC screening for patients with diabetes in the primary care safety-net are needed.

**Methods:**

Guided by the Exploration, Preparation, Implementation and Sustainment Framework, we conducted a stakeholder-engaged process to identify multi-level change objectives for implementing optimized CRC screening for patients with diabetes in FQHCs. To identify change objectives, an implementation planning group of stakeholders from FQHCs, safety-net screening programs, and policy implementers were assembled and met over a 7-month period. Depth interviews (*n* = 18–20) with key implementation actors were conducted to identify and refine the materials, methods and strategies needed to support an implementation plan across different FQHC contexts. The planning group endorsed the following multi-component implementation strategies: identifying clinic champions, development/distribution of patient educational materials, developing and implementing quality monitoring systems, and convening clinical meetings. To support clinic champions during the initial implementation phase, two learning collaboratives and bi-weekly virtual facilitation will be provided. In single group, hybrid type 2 effectiveness-implementation trial, we will implement and evaluate these strategies in a in six safety net clinics (*n* = 30 patients with diabetes per site). The primary clinical outcomes are: (1) clinic-level colonoscopy uptake and (2) overall CRC screening rates for patients with diabetes assessed at baseline and 12-months post-implementation. Implementation outcomes include provider and staff fidelity to the implementation plan, patient acceptability, and feasibility will be assessed at baseline and 12-months post-implementation.

**Discussion:**

Study findings are poised to inform development of evidence-based implementation strategies to be tested for scalability and sustainability in a future hybrid 2 effectiveness-implementation clinical trial. The research protocol can be adapted as a model to investigate the development of targeted cancer prevention strategies in additional chronically ill priority populations.

**Trial registration:**

This study was registered in ClinicalTrials.gov (NCT05785780) on March 27, 2023 (last updated October 21, 2023).

**Supplementary Information:**

The online version contains supplementary material available at 10.1186/s12875-024-02496-0.

## Background

Patients with diabetes mellitus have an estimated 27% elevated lifetime risk of developing colorectal cancer (CRC), and are disproportionately from priority health disparities populations (e.g., low-income, Non-Hispanic Black and Hispanic) [1, 2]. Nationally, guideline concordant receipt of CRC screening for patients with diabetes is not significantly different for women with diabetes (57% vs. patients without diabetes 58%) and is significantly higher among men with diabetes (63% vs. patients with diabetes 58%) [3]. CRC screening for patients with diabetes, who do not have other indications of high risk (e.g., family history of CRC, polyp removal during colonoscopy, personal history of CRC, inflammatory bowel disease) are advised to follow the average risk screening recommendations [4]. Federally qualified health centers (FQHCs) primarily serve as primary care for priority health disparities populations and struggle to sustainably implement CRC screening programs for average-risk patients which includes patients with diabetes. CRC screening uptake in FQHCs populations has been consistently lower (44.1%) than the national average for average risk, age-eligible adults (67.3%) [5].

Persons receiving diabetes care in FQHCs have elevated health risks overall and higher rates of poverty and low-income status than the general population [6]. Ten percent of FQHC patients have a diabetes diagnosis and more than a third within this group have uncontrolled diabetes (HbA1c > 9%). Failure to implement preventive CRC screenings translates to an average of 6.5 years of lost life for patients subsequently diagnosed with CRC [7]. Moreover, this contributes to greater burden for patients with diabetes who are diagnosed with CRC who suffer greater morbidity, all-cause mortality, and cancer-specific mortality compared to CRC patients [8–10]. Therefore, efforts to prioritize CRC screening for patients with diabetes are needed in primary care safety-net settings.

Multiple evidence-based CRC screening tests are available which complicates implementation. The U.S. Preventive Services Taskforce (USPSTF) recommends CRC screening in adults aged 45–75, with multiple screening options available including non-invasive stool based testing: high sensitivity guaiac fecal occult blood tests (gFOBT), fecal immunochemical test (FIT), FIT plus stool DNA testing (FIT-DNA); and direct visualization tests: colonoscopy, computed tomography (CT) colography, and flexible sigmoidoscopy (FS) (with or without FIT) (see Table 1 for intervals) [4]. Colonoscopy and FS, have been shown to reduce mortality by (68% and 28%, respectively). FIT and FOBT are associated with 13–33% mortality reductions. Stool-based testing mortality reductions require sustained annual adherence. [11–14]. Research has shown that failures to screen at all, to screen at appropriate intervals, and to follow-up on abnormal results are associated with risk of CRC death [15].

**Table 1:** Evidence-based CRC tests & testing intervals

Test	Testing Intervals
**Stool-based tests**	
gFOBT	Annual
FIT	Annual
FIT-DNA	1–3 yrs.
**Direct visualization tests**	
Colonoscopy	10 yrs.
CT colonography	5 yrs.
FS	5 yrs.
FS with FIT	10 year/annual

Given major differences in mortality reduction benefits, temporal intervals for retesting, costs, and patient burden, controversies have emerged surrounding the pros and cons of testing methods [16, 17]. Colonoscopy and FS allow for polypectomies, which can prevent CRC [18, 19]; however, FS is not widely used in the U.S, because colonoscopy evaluates the entire colon, can be done every 10 years, and is associated with a greater mortality reduction [20]. A re-analysis of the USPSTF data suggest that prevention, through the removal of polyps during colonoscopy, is the sole mechanism of CRC mortality reductions [19]. Colonoscopy is thus the “gold standard,” despite critiques about the rigor of this evidence (e.g., indirect and observational). [21–24]. In FQHCs, non-invasive tests are emphasized and colonoscopies are often a second line-screening based on abnormal gFOBT/FIT findings. [25]. Non-invasive tests are emphasized because these are less costly, require less time (and time off of work), less complicated to complete, do not require transportation, and are guideline concordant [26]. Despite stool based testing’s acceptability, US-based trials in FQHCs designed to increase annual adherence to stool-based testing have reported low screening adherence over three years (10.4–16.4%) [27–29].

Prioritizing colonoscopy with longer testing intervals in under-resourced FQHCs for patients with diabetes introduces fewer opportunities for care breakdowns, is guideline concordant, and prevents CRC by removing premalignant colonic polyps. Guided by the Exploration, Preparation, Implementation and Sustainment [30]. (EPIS) framework, this research study will develop and evaluate targeted CRCs screening strategies for patients with diabetes in safety-net settings. This study addresses known implementation challenges using a “designing for dissemination” approach [31–33] that attends to important contextual, organizational capacity and patient complexity factors that impact CRC screening program implementation in clinics and uptake among patients with diabetes.

### Conceptual framework

The design of this study was guided by the EPIS framework. EPIS is an evidence-based practice (EBP) implementation framework that includes four defined phases for assessment of inner and outer contextual factors that influence EBP implementation (see Table 2). For this study, the EBP is CRC screening uptake among age eligible patients with diabetes. Exploration is the act of identifying patient needs and the availability of EBPs to address identified needs, and the decision to adopt evidence into practice based on fit within the inner clinical context. During this phase, the adaptations to the evidence are based on system, organization, and individual patient factors. Preparation includes planning implementation, inventorying proposed challenges, and developing strategies to overcome anticipated barriers. A critical component of this phase is the planning of implementation strategies to support EBP utilization in the next two phases and to address organizational climate to ensure that EBPs will be supported, expected, and rewarded. During the clinical trial, this study focuses on implementation, the process of assuring and balancing fidelity to the EBP delivered with adaptations needed to assure program success. Sustainment focuses on maintenance and program and factors impacting implementation over the long haul. EPIS considers innovation factors, which are the characteristics of the EBP being implemented. The innovation-EBP fit considers if the EBP fits the patient, provider, and organizational needs. Innovation factors are assessed and can be adapted to maximize the fit of an EBP while maintaining the core elements of the intervention to retain fidelity.

**Table 2:** EPIS constructs for implementation of SURE: CRC4D toolkit to promote screening in patients with diabetes

Domains	Inner context	Outer context
Actors	Decision makers, Champions, Adopters, Staff	Patients, Policymakers/Advocates, Funders
Resources	Electronic Health Record, Materials, Supports	Referral for CRC screen; Patient Navigation; Community Organizations
EBP	Core elements for fidelity; EBP fit with Organization	EBP fit with practice; Practice Incentives

Acronyms: EBP: evidence-based practice, CRC: colorectal cancer screening

## Methods and design

### Identifying multi-level change levers: a multi-method stakeholder informed approach

Earlier phases of this research focused on the Exploration and Preparation phases, while the current protocol describes the intervention implementation and its evaluation. During the exploration phase, a secondary analysis was conducted of a nationally representative data set to identify patient level determinants of CRC screening uptake overall (i.e., with any test) and test-specific uptake among individuals with diabetes. We explored disparities in uptake overall and testing type based on race, ethnicity, income, and educational status. Additionally, a scoping literature review was performed to identify evidence-based interventions and implementation strategies for CRC screening and diabetes management in FQHCs. Based on this scoping review, we identified additional interventions and implementation strategies, using the Expert Recommendations for Implementing Change (ERIC) taxonomy [34]. A list of interventions and implementation strategies was compiled related to diabetes management processes to expand an existing measure that was developed and used to evaluate the use of evidence-based intervention and implementation for CRC in FQHCs [35].

For the preparation phase of the formative research, we used implementation mapping, an iterative process that incorporates community based participatory research principles [36, 37]. An Implementation Planning Group (IPG) was assembled to represent a diversity of implementation actors (e.g., clinicians, state-level decision makers, screening safety-net programs) who work in and with FQHCs. The goal of the IPG, which met 5 times over a six-month period, was to develop shared understandings of the research problem based on empirical knowledge from the national survey analysis, the scoping review of the literature, and local knowledge of the IPG members about patient population and clinic system capacities. The IPG group identified and prioritized the selection of implementation strategies to improve CRC screening uptake for patients with diabetes. The IPG and research team iterated an implementation plan specifying multi-level change objectives and implementation determinants to develop supports to help prioritize CRC screening implementation for patients with diabetes.

Guided by the insights of the exploration and preparation phases, we developed the Strategic Use of Resources for Enhanced ColoRectal Cancer Screening in Patients with Diabetes (SURE: CRC4D) implementation toolkit, which includes tailorable materials and protocols that will be tested in a single arm, hybrid type 2 effectiveness-implementation single arm clinical trial. The objectives of this trial are to:

1. Determine the effectiveness of the SURE: CRC4D multi-component implementation strategies to increase CRC screening uptake among patients with diabetes.
2. Evaluate the fidelity, feasibility, and acceptability of SURE: CRC4D implementation.
3. Refine the SURE: CRC4D toolkit based on multi-level user feedback and conduct an evaluation to promote scalability and sustainable use.

### Methods

#### Study participants and setting

This single arm trial will be conducted in six FQHC clinical sites in New Jersey. Eligibility criteria for the FQHC clinics include: (1) provide care to at least 450 patients aged 50–74 years; (2) 10% of patient population previously diagnosed with diabetes; (3) located in New Jersey; and, 3) clinical and administrative leadership willing to engage in the intervention and research requirements (interviews, data validation, process evaluation). Implementation outcomes will be assessed using mixed methods guided by the EPIS constructs (see Table 2). The methods of this study have been reported using Standard Protocol Items: Recommendation for Interventional Trials (SPIRIT) guidelines (Supplemental file 1).

During the implementation a clinic-based registry of patients eligible for CRC screening will be developed for each clinic at baseline and updated at six and 12 months post-baseline. Patient eligibility criteria will include: (1) patients not up-to-date or due for CRC screening [4]. based on electronic health record (EHR) documentation (e.g. FOBT/FIT test in last year, flexible sigmoidoscopy within 4 years, or colonoscopy within 9 years), (2) previous diagnosis of type II diabetes, (3) age-eligible for CRC screening (45–74 years of age) and (4) ) FIT/FOBT that has been ordered for more than 6 months that has not been completed or a sigmoidoscopy or colonoscopy referral that has not been completed for 12 or more months. Patients are excluded if they have EHR documentation medical conditions not concordant with standard CRC screening intervals (e.g. prior CRC diagnosis, inflammatory bowel disease, renal failure, etc.) [4].

The NJ Primary Care Research Network (NJPCRN) will recruit eligible clinics for participation. The NJPCRN is an Agency for Healthcare Research and Quality recognized practice-based research in primary care practices. The NJPRN will contact FQHCs that participated in previous research and ask IPG members to make introductions with their FQHC leadership networks. Emails with study flyers will be sent to the FQHC with follow-up telephone outreach. This protocol has been approved by the Rutgers University Institutional Review Board (Pro2020002075). All study participants will be asked to provide informed consent prior to participation in all phase of this research. We expect the distribution of patient participants to reflect the racial/ethnic diversity of the FQHCs recruited, who predominantly serve low-income, racial, and ethnic minority populations.

### Implementation strategies

The goal of SURE: CRC4D is to enable FQHC clinics to adopt strategies to optimize the use of evidence based colorectal cancer screenings (See Table 1) uptake for patients with type II diabetes. To accomplish this, multi-level, multi-component implementation strategies (see Table 3) will be utilized. The core components of this implementation effort includes the identification and engagement of 2–3 clinic change champions, who will participate in two virtual learning collaborative events [38–41] and lead the change effort in the clinic aided by bi-weekly virtual practice facilitator support [38, 42–49]. The SURE: CRC4D toolkit will include guidance on pulling data to develop and implement quality monitoring systems to provide regular audit/feedback to the clinic, patient educational materials in English and Spanish and dissemination materials for clinical meetings to orient other clinic members to the change process being implemented to optimize CRC screening for patients with diabetes [50, 51]. Clinic champions will tailor toolkit resources as clinics may have different electronic medical records, type and composition of staff, clinical workflows, and standing clinical team meetings.

**Table 3:** Timeline for implementation activities & assessment

Single Group	Year in months											
**Implementation Activities**	1	2	3	4	5	6	7	8	9	10	11	12
Identify clinical champions	x											
Learning collaborative	x		x									
Virtual Practice facilitation	x	x	x									
Patient Education Materials	x											
Clinical Meeting Materials	x	x										
Quality Monitoring System	x	x	x	x								
**Assessments**												
EHR abstraction	x											x
Clinic Staff and Clinician Qualitative Interviews	x	x	x			x						x
Practice Information Form	x					x						x
Clinic Staff and Clinician Questionnaires	x					x						x

The implementation will be rolled out over a 12-week period. Initially, clinics will be asked to identify 1–3 clinic champions, with at least one clinician (i.e., physician, advanced practice nurse, physician assistant) per team. Each team will meet with the external practice change facilitator approximately two weeks prior to the initial learning collaborative. This initial facilitation meeting is introductory, with the goal of encouraging clinic champions to reflect about the current clinic CRC screening strategies and diabetes care management processes prior to the 1st virtual learning collaborative. Clinical champions will attend the 1- hour virtual learning collaborative, where the materials in the SURE: CRC4D toolkit will be provided and reviewed, and each clinic team will formulate practice change goals. Teams will decide on how to deploy the toolkit strategies at their FQHC sites over the course of the next ten weeks. The practice facilitator will support the clinic champion team in the development, implementation, and refinement of the local practice change plan. The champions will meet with the practice facilitator every two weeks for 8 weeks (4 times). During this time, the plan will be refined and adjusted based on feedback from clinic leaders and practice staff members and identified strengths and barriers that are encountered during the implementation effort. At week 10, a second learning collaborative will be virtually convened, providing a forum where the different clinic teams can share their successes and obstacles during the development and execution of their plan. This forum will foster cross-team learning and idea generation that can inform the refinement of the SURE: CRC4D toolkit and sustainability of practice change efforts. Two weeks after the second learning collaborative, a final virtual facilitation meeting will be held to reflect and refine the practice plan to support sustainability.

### Evaluation of the effectiveness and implementation of SURE: CRC4D

The effectiveness and implementation of SURE: CRC4D will be evaluated using a mixed method learning evaluation strategy, where ongoing data collection and analysis are used to refine implementation to optimize adoption of CRC screening for patients with diabetes [52, 53]. This evaluation is designed to address two research questions: (1) are the adapted implementation strategies clinically effective in increasing CRC screening rates for patients with diabetes; and, (2) are the implementation strategies feasible and acceptable to implementers (e.g., clinicians and staff) and patients in FQHCs? This evaluation builds an evidence base about the effectiveness of the implementation strategies in a real-world context and allows for the collection of data that can be used to refine the implementation toolkit for a larger scale, definitive cluster randomized controlled trial. Guided by EPIS, contextual factors were selected based on suggestions from clinical stakeholders, community partners, and previous literature suggesting they may influence implementation success [54–56] (see Table 4). The following assessments and measures will be collected to evaluate the trial:

**Table 4:** Study measures

	Baseline & 12-month post implementation
Effectiveness: Clinic Level Patient Outcomes*	
Demographics	QUANT
Glycemic Control (HgbA1C < 9)	QUANT
Any CRC screening/patients eligible	QUANT
Colonoscopy completion/patients eligible	QUANT
CRC rate by glycemic control	QUANT
**Contextual Factors: Clinic and Clinic Team-Level Variables**	
Implementation Climate/History of Implementation^36^	QUANT
Change Process Capability	QUANT
Adaptive Reserve	QUANT
Medical Provider/Staff Demographics (e.g., age, sex, race, training, years in practice)	QUANT
Facility Resources	QUANT/QUAL
Organizational Readiness for Change^84^	QUANT/QUAL
Implementation Leadership Scale/Leadership Style	QUANT/QUAL
Implementation Outcomes*	
Patient Acceptability	QUANT
Staff/Clinician Fidelity	QUANT
Staff/Clinician Acceptability	QUAL
Feasibility	QUAL

*Note* * These assessments will be collected from the electronic health record in aggregate at baseline and 12 months post implementation; QUANT = quantitative; QUAL = qualitative

### Organizational assessments

Guided by EPIS, contextual factors will be evaluated at baseline and 1 year-post implementation. Medical Directors or the Chief Operating Officer of each clinic will be asked to complete a web-based survey called the Clinic Organizational Information Form (COIF). This survey assesses *Implementation Climate* and *History of Implementation* related to CRC screening and diabetes management [35]. Additionally, patient demographics, management strategies, and payor mix are collected using this survey for each clinic.

### Clinic staff measures and assessments

The Clinic Staff Questionnaire (CSQ) will be administered to all practice clinicians and staff members at baseline and 12-month post-implementation. The clinic team measures include *Medical Provider and Staff Background* and history with the organization. Additionally, *Change Process Capability* will be measured, specifically “previous history of change,” and “ability to initiate and sustain change.” [57]. These two measures have been identified these as key mechanisms for successful organizational change and its wide use in cardiovascular care implementation [58–60]. Additional practice-based measures will include: *Adaptive Reserve* a feature of resilient organizations shown to be associated with practice-level implementation of CRC screening, will be measured in the CSQ with the validated 23-item scale [57, 61]. The CSQ will also include the *Implementation Leadership Scale* (ILS), a brief psychometrically strong measure that contains 12-items with four subscales of proactive, knowledgeable, supportive, and perseverant leadership [62].

### Process data outcomes

Learning collaborative and facilitation phone calls will be audio recorded and transcribed to document issues that arose during the implementation process. Additionally, qualitative interviews will be conducted at baseline and beginning at 6 months post implementation. We will select key implementers (3–4 individuals per site) to assess perceptions of organizational readiness to change, leadership style and additional facility characteristics (e.g., assets and deficits of location, satisfaction with ease of access to facility, etc.). Staff and clinician perceptions of the SURE: CRC4D implementations’ feasibility and acceptability will also assessed asking providers and staff to describe their implementation experiences. The interviews will probe stakeholder perceptions of change in their organizations, systems, and factors that they think impacted implementation. Staff or provider fidelity will be assessed based on the clinic-level proportion of eligible patients who were (1) contacted based on implementation protocol and (2) completed any CRC screening at 1 year.

### Patient level: clinical effectiveness outcome and implementation assessment

The primary outcome variables to assess clinical effectiveness will be the clinic level proportion of patients with diabetes who: (a) receive any CRC screening and (b) complete a colonoscopy at 12 months from baseline. An exploratory analysis will assess clinic-level CRC screening completion by glucose control (controlled vs. uncontrolled, i.e., HbA1c > 9 at 12 months). Patient level data is collected in aggregate and will include no identified personal health information.

Patient acceptability will be assessed through the assessment of patient rates of opting-out and non-adherence of CRC screening. This rate will be based on the proportion of CRC screening among patients with diabetes compared to overall eligible patient population (without diabetes) in each clinic.

### Data analyses

#### Qualitative analysis

On a quarterly basis, we will analyze data from each clinic site using a comparative case analysis [63]. Organizational level data and interview transcripts will be organized, read and coded in ATLAS.ti. Data will be analyzed on an ongoing basis, and a working summary of emergent findings will be updated as incoming data is added. As a validity check of qualitative results, we will check relevant data interpretations against all new data using a constant comparison approach [64]. We will note similarities and differences of implementation feasibility between practice sites based on clinic characteristics and from data provided in interviews. Each quarter all quantitative and qualitative results will be summarized in brief reports to be shared with the research team for reflections on any changes needed. These analyses represent ongoing monitoring and feedback to inform refinements of the implementation strategy and clinical trial procedures to refine implementation strategy to better fit local needs and contexts.

### Quantitative analysis

Descriptive statistics will be used to summarize patient and clinic characteristics. We will declare our intervention a success if at least 25% of those unscreened are screened at 12-month follow-up in this difficult to reach population. We will declare the optimization of screening a success if 15% of those unscreened are screened with a colonoscopy or flexible sigmoidoscopy at 12-months. Overall improvement metrics are comparable to improvements in previous CRC screening implementation studies in FQHCs [65, 66]. At baseline, we will calculate average screening rates and their confidence intervals across all practice sites in intent to treat analyses and at 12-months we will assess screening rates and their confidence intervals for all sites. The confidence intervals will be compared to 25%. We will compare differences in CRC screening by glucose control, sex, and race/ethnicity.

### Power calculations

The value of information method [67] was utilized to select a sample size balancing the costs and feasibility goals of the trial. This sample size (e.g., six clinic sites, assuming at least *n* = 30 patient in each) is sufficient to generate preliminary estimates of the estimated effect (80% confidence interval) of the implementation strategy on CRC screeningrates [68, 69]. In developing the power calculation, we assume equal numbers of patients (*n* = 50) per clinic (the anticipated number of eligible patients, *n* = 450 CRC screening eligible, with > 10% diabetes diagnosis). Of those with diabetes, we expect 40% to be up-to-date with screening guidelines based on the average rate of CRC screening in FQHCs [70]. Thus, the target sample size is *n* = 30 patients in each FQHC.

## Discussion

This study aims to optimize CRC screening using the engagement of multi-level stakeholders (patients, clinicians, staff in FQHCs) and using an implementation mapping during the exploration and preparation phases prior to implementation [37]. This project is innovative in several key ways. Regarding conceptual innovation, few studies have included CRC screening as a component of diabetes care prior to CRC diagnosis [71, 72], while many have focused on improving CRC screening for average risk adults in FQHCs [65, 66, 73–79].An EPIS framework systematic review concluded that attention to planning EBP use is “infrequent though critical [80].” FQHC implementation of CRC screening programs focus on achieving the Uniform Data System (UDS) targets, which do not distinguish patients at greater risk for CRC in the “average-risk” patient population [70, 81]. Metrics for UDS CRC screening program are also cross-sectional and collected as separate metrics unrelated to diabetes care or annual stool-based testing adherence. For FIT and FOBT stool-based CRC screening strategies to be clinically effective and for their mortality reductions to be realized sustained annual adherence is required, which has been proven difficult to accomplish in safety-net primary care settings [12, 13]. Additionally, few FQHCs formally assess factors related screening prior to implementing improvement interventions [82]. This study aims to optimize CRC prevention using the engagement of multi-level stakeholders (patients, clinicians, staff in FQHCs) and using an implementation mapping during the exploration and preparation phases prior to implementation [37].

Despite being the most studied evidence-based cancer screening in the National Institutes of Health implementation science portfolio, no systematic studies have integrated CRC screening and diabetes evidence-based approaches to prioritize preventive care for patients with diabetes in the primary care safety-net. To date, research has focused on overall CRC guideline adherence, relying on an ‘all boats rise’ approach despite the failures of such strategies to achieve improvements in chronic disease targets [83]. In contrast, this study focuses on optimizing CRC screening using targeted implementation strategies to address disparities among individuals with diabetes to promote health equity.

Study findings are poised to inform the develop scalable, equitable approaches to CRC screening in safety-net primary care settings. If successful, next steps will include testing the scalability and sustainability in federally qualified health centers nationally. Further, this approach can be adapted as a model to investigate the development of targeted cancer prevention strategies in additional chronically ill priority populations.

### Electronic supplementary material

Below is the link to the electronic supplementary material.

Supplementary Material 1

## Data Availability

No datasets were generated or analysed during the current study.

## Abbreviations

CRC: Colorectal cancer
CT: Computed tomography
FIT: Fecal immunochemical tests
FIT-DNA: Fecal immunochemical test stool DNA testing
FQHC: Federally qualified health centers
FS: Flexible sigmoidoscopy
gFOBT: Guaiac fecal occult blood tests
IPG: Implementation Planning Group
HER: Electronic health record
EBP: Evidence-based practice
ERIC: Expert Recommendations for Implementing Change
EPIS: Exploration, Preparation, Implementation, Sustainment
NJPCRN: New Jersey Primary Care Research Network
SURE: CRC4D: Strategic Use of Resources for Enhanced ColoRectal Cancer Screening in Patients with Diabetes
SPIRIT: Standard Protocol Items: Recommendation for Interventional Trials
UDS: Uniform Data System
USPSTF: U.S. Preventive Services Taskforce
